# Clinical features for diagnosis and management of patients with PRDM12 congenital insensitivity to pain

**DOI:** 10.1136/jmedgenet-2015-103646

**Published:** 2016-03-14

**Authors:** Stella Zhang, Saghira Malik Sharif, Ya-Chun Chen, Enza-Maria Valente, Mushtaq Ahmed, Eamonn Sheridan, Christopher Bennett, Geoffrey Woods

**Affiliations:** 1 School of Clinical Medicine, University of Cambridge School of Clinical Medicine, Cambridge, UK; 2 The Yorkshire Regional Genetics Service, Chapel Allerton Hospital, Leeds, UK; 3 Cambridge Institute for Medical Research, University of Cambridge, Cambridge Biomedical Campus, Wellcome Trust, Cambridge, UK; 4 Section of Neurosciences, Department of Medicine and Surgery, University of Salerno, Italy

**Keywords:** neuropathy, insensitivity to pain, staphylococcus, charcot's joints, keratitis

## Abstract

**Background:**

Congenital insensitivity to pain (CIP) is a rare extreme phenotype characterised by an inability to perceive pain present from birth due to lack of, or malfunction of, nociceptors. *PRDM12* has recently been identified as a new gene that can cause CIP. The full phenotype and natural history have not yet been reported.

**Methods:**

We have ascertained five adult patients and report their clinical features.

**Results:**

Based on our findings, and those of previous patients, we describe the natural history of the PRDM12-CIP disorder, and derive diagnostic and management features to guide the clinical management of patients.

**Conclusions:**

PRDM12-CIP is a distinct and diagnosable disorder, and requires specific clinical management to minimise predictable complications.

## Introduction

Pain is a sensory modality present in all complex organisms and used to detect potential and real tissue damage.^1^ ^2^ Although it is an unpleasant sensory and emotional experience, it substantially affects our behaviour, providing a survival advantage.^3^

Normally, pain is detected by a complex system of mechanical and chemical sensors called nociceptors, which then send information through spinal interneuronal pathways to the brain.^4^ ^5^ However, in a number of rare conditions, components of the pain-signalling pathway may be impaired or fail to develop.^6^ One such condition is congenital insensitivity to pain (CIP), where individuals are unable to perceive pain from birth.^7^

There are two common forms of CIP. First, loss-of-function mutation in the *SCN9A* renders nociceptors unable to respond to any noxious stimulus.^8^ Second, loss-of-function mutation in the *NTRK1* leads to a failure of nociceptors to develop.^9^ Recently, a new form of CIP has been described, caused by mutations in an epigenetic regulator *PRDM12*.^10^ ^11^ The phenotype of these patients was briefly annotated in the initial study. Here, we describe the clinical features of five adult patients with PRDM12-CIP, derive diagnostic criteria for this specific form of CIP and suggest management guidelines.

## Method

We studied five adults diagnosed with a PRDM12-CIP. Four were male and one female, and their ages were 23, 30, 42, 44 and 57 years. Four of the five were from different branches of the same consanguineous family; two siblings and two cousins. All had homozygous mutations that had been proven to be pathogenic, missense or expansion of the C-terminal *PRDM12* polyalanine tract.^10^ Research ethics approval had been granted for the study.

Each of the five adult patients was seen by one of the authors, a history and pedigree taken, examination performed, their physician contacted and medical notes scrutinised. Subsequently, we conducted a detailed telephone interview using a qualitative interview questionnaire, see online supplement. On both occasions, the patients were either seen with their parents, or had discussed the relevant questions with their parents. Prior to conducting the interview, informed consent was obtained for the study from all five adult patients. The set of questions were then administered through a phone interview, see online supplement.

10.1136/jmedgenet-2015-103646.supp1 Supplementary data

## Results

Here, we describe the key clinical features of patients with PRDM12-CIP, and the corresponding implications for diagnosis and management. As four of the individuals had the same mutation, the consistency of phenotypic features may be overestimated.

### Insensitivity to pain

In keeping with the phenotype of CIP, all individuals are unable to sense acute pain or chronic pain. While this was present from birth, CIP was only diagnosed after the age of three. Due to their inability to sense pain, all five individuals have sustained a large number of unusual self-inflicted injuries. Three individuals had lost their tongue tip due to autoamputation in childhood as well as injuries to their lips, tongue, gums and the inside of their cheeks.^12^ Three individuals had sustained fractures of the upper and lower limbs.

However, unlike SCN9A and NTRK1 CIP, patients with PRDM12 mutations could experience non-global pain insensitivity. In one individual insensitivity was restricted to their limbs and head; in another individual pain insensitivity was complete in the left leg, but partial in the right.

### Other pain-related features

Staud proposed that individuals who are insensitive to pain might be deficient in multiple different components of the ‘pain’ experience.^13^ PRDM12-CIP individuals were able to taste types of food that are commonly associated with a painful sensation; being able to recognise spicy foods as being ‘hot’. They were able to feel a full normal range of emotions, including painful emotions. SCN9A and NTRK1 CIP individuals are also able to recognise a hot taste and to feel emotional pain.

### Normal neurological examinations and intellect

All five individuals were of normal intellect and had a normal neurological examination, including fine touch, deep touch, pressure, vibration and sensation of itch and tickle. Olfaction was also normal, in contrast to the anosmia of individuals with SCN9A CIP.^14^

### Temperature sensing and thermoregulation

All individuals reported being able to sense gross temperature differences/ranges (separate to their inability to recognise painful extremities of temperature). However, they were unable to identify noxious stimuli—for example, if food was too hot—and thus at risk of heat or cold injuries. Four individuals reported normal sweating and normal thermoregulation. One individual could not sense ambient temperature, and so had encountered difficulties with hyperthermia and hypothermia. A clinically significant inability to sense temperature is also a feature in NTRK1 CIP, but temperature sensing is normal in SCN9A CIP.

### Corneal abrasions due to lack of tear production

All individuals reported impaired tear production, and on examination had an absent corneal reflex. This resulted in a significantly increased risk of corneal injuries, and thence keratitis and corneal scarring—more so than any other form of CIP. Two individuals had no useful vision in one eye, and in one multiple corneal grafts had failed.

### Recurrent infection

All individuals reported recurrent infections with unknown causes and sudden onset overnight. These tend to occur in their feet and hands, and there was no obvious accident or cause for these infections that the individuals could recall. One required a below-knee amputation of his right leg to treat a serious ongoing chronic infection that had not responded to therapies. Another developed a paraspinal abscess that required surgical intervention for its resolution. Infections were not painful, and inflammation was always far less than expected by doctors. All had lost terminal digits through minor injuries becoming chronically infected, and then progress to osteomyelitis, which was often only treatable by amputation. Interestingly, none had suffered from septicaemia or pneumonia. *Staphylococcus aureus* caused all significant infections.

Deleterious mutations of another gene associated with a painless phenotype, NTRK1, are also associated with recurrent *S. aureus* infections. NGFβ-TRKA signalling has been found to have a role in innate immune signalling.^15^ Why PRDM12-CIP should predispose individuals to recurrent infections is unknown.

### Charcot's joints

Surprisingly, none of the individuals described were thought to have significant Charcot's joints, either by symptoms or by radiographic examination. Why this should be is puzzling, as all individuals with SCN9A and NTRK1 CIP will have at least one Charcot's joint by mid teenage years, and have multiple affected joints after the age of 20 years.^16^

## Discussion: diagnosis and management

### Considering CIP as a diagnosis

CIP should be considered as a diagnosis in any child presenting with a history of poor or absent responses to painful stimuli. CIP often presents with unexplained oral injuries (especially NTRK1 and PRMD12 CIP), unexplained burns, bruises, fractures and joint injuries; as described in the Results. External examination may reveal an excess of injuries, and there is a potential for confusion with neglect and with child abuse. A simple pressure test (pen pressed onto the nail bed with 5–10 kg force) will usually reveal those who feel no pain, by the lack of a withdrawal response.

Neurological examination is normal, but skin biopsy (seeking a loss of bare nerve termini in the epidermis) or nerve biopsy (seeking the deficiency of small myelinated nerve fibres unique to this condition) can be diagnostic, but is uncommon in current clinical practice.

### Distinguishing PRDM12-CIP

Of the possible genetic causes of CIP, PRDM12-CIP should be considered if painlessness is congenital, if it is of a non-global nature (but this is not obligatory, however it is unreported in other forms of CIP), if intellect is normal, sense of smell is normal (difficult to test reliably <5 years) and if corneal abrasions are detected in excess or at an early age (<8 years). Table 1 summarises the key clinical features that distinguish SCN9A, NTRK1 and PRDM12-CIP.

**Table 1:** Summary of differences between painlessness caused by SCN9A, NTRK1 and PRDM12 mutations

	Congenital insensitivity to pain		Congenital insensitivity to pain with anhidrosis
Gene	SCN9A	PRDM12	NTRK1
Inheritance	Autosomal recessive	Autosomal recessive	Autosomal recessive
Pain	Impaired ability to feel pain		
Autonomics	Sweat: normal Lacrimation: normal	Sweat: normal Lacrimation: reduced	Sweat: anhidrosis, recurrent febrile episodes, hypothermia in cold Lacrimation: may be reduced
Cranial nerves	Smell: anosmia	Smell: normal Corneal reflexes: may be absent Recurrent *Staphylococcus aureus* infections	Smell: normal Corneal reflexes: may be absent Recurrent *S. aureus* infections
Peripheral nerves	Inability to sense pain	Inability to sense pain	Inability to sense pain and temperature
CNS	Normal intellect, otherwise normal development		Mental retardation, variable

CNS, central nervous system.

### Management of ophthalmological complications

Individuals with PRDM12-CIP appear to have a greater incidence of corneal abrasions than other types of CIP. Management includes at least yearly ophthalmological assessments, regular use of eye lubricants during the day and at night, eye protection in windy or dusty conditions and the avoidance of irritants and chemicals (eg, bleach, onion and fumes).

### Management of infections

Patients are at increased risk of repeated Staphylococcal infections that arise without an obvious external cause, see Results. The early use of topical antibacterial creams until all signs of inflammation or infection have resolved was reported to be highly effective in preventing chronic infection. For more significant infections, the lack of pain as a sign had hindered the diagnosis of osteomyelitis and septic arthritis, and all involved physicians should be aware of this. Most significant infections were complicated and required a course of IV antibiotics before they resolved.

### Management of injuries

As patients with CIP are unable to feel pain, this impairs their ability to detect injuries promptly and appropriately immobilise the injury to allow for healing. To encourage prompt detection of injuries, patient/parents should conduct daily self-checks particularly of vulnerable regions such as their feet, hands and joints. The medical management of any injuries is as for other forms of CIP. Currently, an annual MRI skeletal survey is not recommended for PRDM12-CIP, whereas it is in SCN9A CIP, because of the lack of proven Charcot's joints in PRDM12.

## Conclusion

PRDM12-CIP is a phenotypically distinct form of ‘Congenital Insensitivity to Pain’; early evidence of corneal scarring, the preservation of some pain sensing in some individuals, normal intelligence, a normal sense of smell and sweating being present (although reduced compared with unaffected siblings). Management is lifelong with the parents, and later the individual, displaying constant vigilance for signs of corneal damage, infections (which are usually *S. aureus*, and must be treated aggressively) and bone/joint injury.
