# Supplementary material for: Clinical features for diagnosis and management of patients with PRDM12 congenital insensitivity to pain
Source: J Med Genet. 2016 Mar 14;53(8):533–5. doi: 10.1136/jmedgenet-2015-103646 (PMC4975812; doi:10.1136/jmedgenet-2015-103646)
Supplement: Supplementary data [file jmedgenet-2015-103646supp.pdf]

Supplement.

Questions asked during the telephone questionnaire session.
These questions focused on the following areas:

- Initial symptoms and diagnosis
- Past medical history
- Current physical health
- Sensory modalities
- Challenges faced by patients
- Strategies employed by patients and healthcare professionals in the management of their condition

HOW DIAGNOSED
Could you please start at the beginning and describe what were the first things you noticed about yourself – on hindsight – that were the first symptoms of painlessness?
Age first noticed symptoms

- Initial symptoms
- What was it that led you to first see a doctor about this?

Age of diagnosis

- When were you first diagnosed?
- What was the route through health practitioners that you took to get this diagnosis?

PHYSICAL ASPECTS
During childhood

- Do you remember much about your experiences in childhood?
- How did you behave during immunisations?
- How did you behave during blood tests (heel prick test)?
- How were you when you were teething?
- How were you when you were learning to walk?
- How is your movement now? – clumsy, heavy handed, heavy footed?

Vision

- How is your vision?
- Do you have any problems with your eyes? Have you had any injury to your eyes?
- Do you use any special protection for your eyes?

Taste

- Do things like onions, red chillis, curries, spices or menthol have an effect on you?
- For instance, do you cry when you cut onions? Do you feel anything when you get chilli in your eye? Do you feel anything when you eat very spicy food? → tearing

Mouth

- Problems with teeth – health of teeth, teeth pain, gum disease, loss of teeth, when lost teeth
- Lips, tongue, inside of mouth – ulcers, scars, injuries

Musculoskeletal

- Soft tissue injuries – cuts, burns, bruises, abrasions, scrapes
- Fractures, joint injuries
- (Tell me about these)
- Did you notice it at the time? When did you first notice it?
- How many times has it happened?

- Complications, treatment, sequelae, recurrence etc.

Infections

- (Tell me about these)
- Recurrence, complications, frequency, treatment, sequelae etc.

Other senses

- Smell: Have you noticed anything about your sense of smell? Would you say that you are able to smell the normal range of smells?
- Numbness: Has you ever felt a “dead arm”? Do you get “pins and needles”? Do you feel “numbness”?
- Itchiness: Do you feel “itchiness” and does it bother you? (e.g. bug bites, chicken pox)
- Temperature: How can you tell if something is too hot or too cold? Can you identify if drinks are too hot or ice cubes? Have you ever had a problem with that?
- Emotion: Whilst not being able to feel pain in the conventional/literal sense, are you able to feel emotional pain?
- Ill: Do you feel ill when you gets an infection or after an injury – the same way that other people feel poorly or sickly, do you feel that?
- Autonomics: Are you able to sweat? Where do you sweat? – what happens when you feel too hot or too cold? How do you feel?

Medical treatment

- Have you ever had any sort of surgery? Were you in pain during the procedure? Did you need anaesthetics? Were you in pain after the procedure?
- How do doctors treat you?
- How do dentists treat you?
- Do you think this is different (for better or for worse) because you are unable to feel pain?

BEHAVIOURAL ASPECTS

- Although you are unable to feel pain, is there any specific behaviour that you’ve adopted in response to pain? How has this changed as you have grown up (e.g. learned pain behaviours)?
- Would you say that your behaviour has been affected as a result of not being able to feel pain (e.g. behaving more recklessly or dangerously)?

BROADER FAMILY EXPERIENCES

What sort of impact has your condition had on your life?

What sort of impact has your condition had on your family life and other relationships?

Were there any previous allegations of child abuse?

Have you told any one else about your diagnosis? How did they react? What do they say?

LIFESTYLE AND MEDICAL MANAGEMENT

Management by family

- Do you have any system of noticing injuries? How do you manage your health?
- Is there anything you have tried to prevent yourself from being injured etc?

Management by medical professionals

- How has your condition been managed by medical professionals?

- Has anything been put into place to support you medically? (e.g. check-ups, medical alert bracelet, letters)

Management by others

- How has your condition been managed by her school?

Suggestions

- Is there anything you want medical professionals to be aware of when caring for patients who can’t feel pain? Is there any way you think doctors can best care for patients like you?
- Is there anything in particular you’ve noticed that might be useful for us when trying to identify patients who can’t feel pain? For instance chewing on their lips or tongue.
